# Development of a Flexible Parallel Wire Robot for Epicardial Interventions

**DOI:** 10.1002/rcs.70130

**Published:** 2026-01-06

**Authors:** Aman Ladak, Johannes O. Bonatti, Roger J. Hajjar, Alaaeldin A. Shalaby, Cameron N. Riviere

**Affiliations:** ^1^ Department of Mechanical Engineering Carnegie Mellon University Pittsburgh Pennsylvania USA; ^2^ Department of Cardiothoracic Surgery University of Pittsburgh Pittsburgh Pennsylvania USA; ^3^ Gene and Cell Therapy Institute Mass General Brigham Boston Massachusetts USA; ^4^ Department of Medicine University of Pittsburgh Pittsburgh Pennsylvania USA; ^5^ The Robotics Institute Carnegie Mellon University Pittsburgh Pennsylvania USA

**Keywords:** beating‐heart surgery, cable‐driven parallel robots, epicardial intervention, gene therapy, robotic surgery, subxiphoid access

## Abstract

**Background:**

HeartPrinter is a flexible parallel wire robot that adheres to the beating heart with vacuum suction at three bases. An injector head actuated by cables delivers gene therapy injections within the bounds of the bases. To deploy onto the epicardium, an introducer mechanism is required. On the heart, the robot's workspace and anatomical model registration to its pose are needed.

**Methods:**

We present HeartPrinter's components and introducer mechanism, and assess them on an artificial beating heart. We evaluate accuracy for position determination of the bases and registering a three‐dimensional heart scan.

**Results:**

The introducer mechanism successfully positioned HeartPrinter, and the bases adhered to the beating heart. The base positions and registration were calculated accurately with errors under 4 and 2 mm.

**Conclusions:**

The introducer mechanism can deploy HeartPrinter on the epicardium, and HeartPrinter's components can operate on the heart. Workspace determination and registration demonstrate feasibility as preliminary concepts.

## 1 Introduction

The field of clinical gene therapy has advanced significantly in both regulatory and commercial aspects [1]. However, progress in cardiac gene therapy has stalled [2], primarily due to inadequate gene delivery mechanisms to the myocardium [3, 4]. Despite ongoing trials and advancements in identifying signalling pathways and vector creation [3], current delivery methods fail to achieve sufficient therapeutic expression in myocytes for effective heart disease and heart failure treatment [4].

Gene therapy serves as a targeted treatment designed to correct mutated genes or specific alterations linked to disease prevalence [4, 5]. Recombinant adeno‐associated virus vectors, which lack viral DNA, are particularly attractive for gene therapy as they can traverse the cell membrane and deliver their DNA cargo into the nucleus of a cell [6, 7]. Myocytes, being primarily non‐dividing cells, are ideal targets for adeno‐associated virus vectors in cardiac gene therapy [6]. A notable application is modulating atrioventricular nodal conduction to regulate heart rate by altering target cells [8].

Various delivery methods have been developed for cardiac gene therapy, such as aortic cross clamping, intravenous injection, and antegrade coronary artery injection [3]. However, such mechanisms have not performed well [8], demonstrating insufficient levels of transduction efficiency and uniformity [3]. Direct gene injection into the myocardium has demonstrated potential [4, 9]; however, current instrumentation only allows a small area of the myocardium to be reached [6]. This results in gene expression over a limited region, representing a lack of diffusion of the vector genome into myocytes [6, 9] and insufficient therapeutic response [9]. An optimal gene delivery method must ensure that the proportion of targeted tissue is reflective of the disease application [3]. For most arrythmia and heart failure applications, effective direct injection requires numerous small and accurate injections over a large area of the myocardium [10]. As this produces dense and homogenous gene expression at the target site [11], the morbidity of cardiopulmonary bypass could also be avoided if these injections are delivered while the heart is beating [12, 13].

Cardiac interventions have shifted towards minimally invasive procedures, reducing patient recovery times, risk of infection, and scarring [14, 15]. With minimally invasive thoracoscopic techniques, however, which utilize rigid tools, the surgeon's workspace is diminished, which restricts the efficacy of gene therapy delivery with direct injection to the myocardium [6]. Alternatively, robotic minimally invasive surgery is a feasible option due to increased access, repeatability, and dexterity [15, 16, 17, 18]. However, challenges remain in adapting robotic systems to the beating heart [16, 19, 20], particularly due to limited thoracic space [21] and the complexity of motion production in the presence of arrhythmias [22].

**FIGURE 1:**
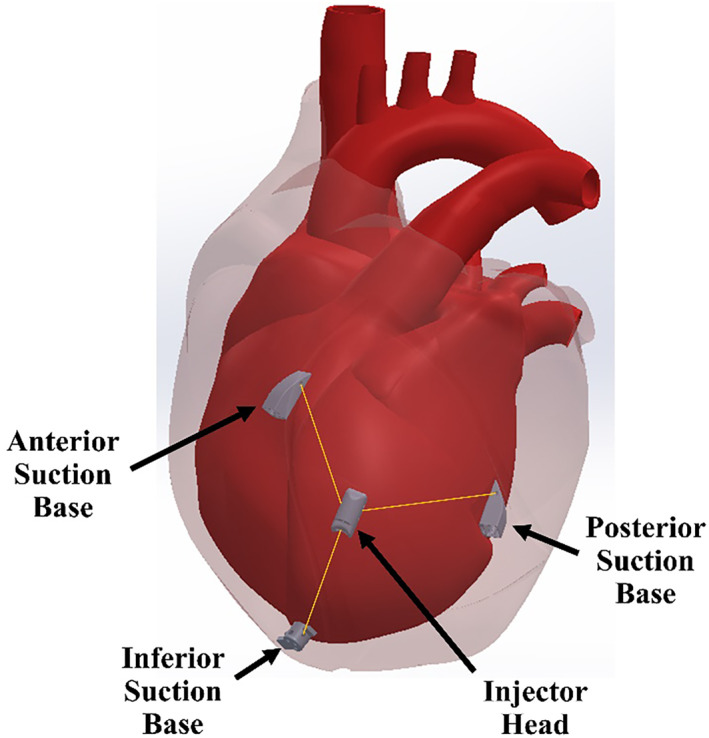
Depiction of HeartPrinter positioned on the region of the epicardium defined by the left ventricle, under the pericardium. The suction bases, injector head, and cables that connect to the injector head are shown. For simplicity, the suction lines and cables exiting the subxiphoid access port are not shown.

HeartPrinter, shown in Figure 1, is a robotic manipulator designed for minimally invasive cardiac gene therapy injections [13]. As a flexible parallel wire robot, it delivers precise injections across the myocardium of the beating heart, offering the potential to avoid the portion of morbidity that results from cardiopulmonary bypass. While we refer to it as a ‘robot’ throughout this manuscript, it is more accurately characterized as a cable‐driven parallel manipulator that performs targeted motion tasks rather than exhibiting fully‐autonomous behaviour. HeartPrinter's primary design requirements focus on its insertion, operation and removal. The robot must be deployable via a subxiphoid approach, navigate under the pericardial sac, and position itself on the epicardium. Once deployed, the system must passively compensate for cardiac and respiratory motion while rapidly transversing the epicardium to deliver precise injections. Minimizing weight and enhancing flexibility will further optimize HeartPrinter's performance and efficiency. After injection delivery, the robot must be easily removed from the patient to minimize procedure time and injury risk.

**FIGURE 2:**
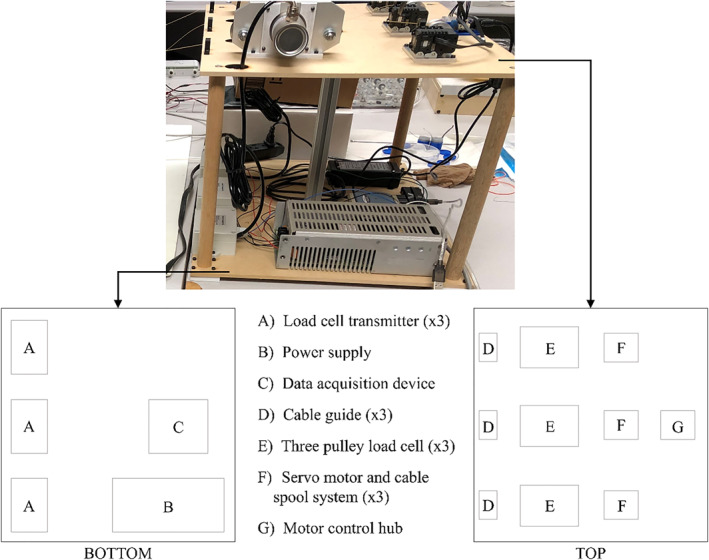
Tabletop instrumentation for HeartPrinter. The actuators, which control the cable length between the injector head and suction bases are shown. Various other components are shown, such as the load cells and data acquisition device, which are used to measure the tension in each cable.

Using an introducer mechanism [23], the robot is inserted via a subxiphoid approach into the pericardial space and deployed on the epicardium. The robot stabilizes itself on the beating heart with vacuum pressure at three suction bases, passively compensating for cardiac and respiratory motion [23, 24]. This eliminates the morbidity associated with arrested heart surgery, such as neurologic dysfunction and major vessel damage [21], and eliminates the need for lung deflation [23]. Within the triangular workspace formed by the suction bases, an injector head can move along the epicardium via three cables that attach to its sides [24]. These cables pass from the injector head through the suction bases and exit the patient through the subxiphoid access port, where they are controlled by actuators in a tabletop setup adjacent to the patient, shown in Figure 2. The actuators control the injector head's position by changing cable lengths between the injector head and the respective suction base [25, 26]. This cable‐driven design allows HeartPrinter to administer many accurate direct gene injections rapidly over a large region of the beating heart [26]. The region of the epicardium defined by the left ventricle is targeted for injection, as preserving its function is a key focus of ongoing gene therapy research [27].

As HeartPrinter's end effector is controlled by flexible cables, this makes it ideal for tasks requiring flexibility over a large workspace [28]. Cable‐driven parallel robots offer low inertia and a high payload‐to‐weight ratio, enabling swift operation of the end‐effector to operate swiftly within its workspace [28, 29]. As an under‐constrained system with three cables and three degrees of freedom [26], HeartPrinter relies entirely on its tabletop actuators to maintain cable tension [30], as the cables can only exert tensile forces [31].

This flexibility of HeartPrinter, however, makes intrapericardial deployment challenging after insertion into the pericardial space [23], particularly in positioning the suction bases on the epicardium anteriorly, posteriorly, and near the apex of the heart. Initial designs lacked compatibility with a positioning mechanism and were relatively large, limiting the extent of minimal invasiveness. The surgical workflow, including determination of the workspace and registration of the pose of the robot, along with the analogous software pipeline, had yet to be developed for HeartPrinter.

This paper addresses these needs by presenting significant new development of the HeartPrinter system from a hardware and software standpoint. Firstly, the development of HeartPrinter's components for operating on the epicardium and the tabletop hardware setup are presented, which reduces the footprint of the robot and ensures compatibility with the introducer mechanism for deployment on the heart. Secondly, further development of the introducer mechanism is shown through design, prototyping, and testing, building on earlier concepts [23]. The updated mechanism includes specialized tools to gain and maintain access in the intrapericardial space, and a steerable mechanism is used to guide the suction bases to their respective target regions. Evaluation of the new design of HeartPrinter and the introducer mechanism is performed on a rubber beating‐heart model using a subxiphoid approach.

Once HeartPrinter is deployed on the heart, the robot follows a routine to define its workspace boundaries and register the preoperative anatomical heart model to the pose of the robot. In this work, determination of the robot's workspace and registration of its pose are formulated within the pericardial sac on a static, non‐beating heart with an uneven surface geometry and increased texture, while excluding the thorax. The irregular surface features and higher friction within the pericardial sac substantially hinder manoeuvrability, as the small components of HeartPrinter tend to become entangled in the surface's bumps and dips. This work builds upon the most recent HeartPrinter study [25], which was conducted on a 3D‐printed prolate ellipsoid with a uniform surface geometry.

## 2 Materials and Methods

This study did not involve human participants, animals, or sensitive data requiring ethical approval. Therefore, no ethical review was necessary. The present prototype uses 3D‐printed and non‐biocompatible materials solely for benchtop evaluation. Because HeartPrinter is not implanted and would interact with the epicardial surface only transiently during a surgical procedure, long‐term biocompatibility testing is not applicable at this stage. Similarly, arrhythmia induction or management was not assessed, as the study did not involve in vivo electrophysiology. Future preclinical and clinical development will require redesign using medical‐grade materials, appropriate biocompatibility testing, and integration with standard intraoperative cardiac monitoring and arrhythmia‐management protocols.

The Materials and Methods section is organized into Sections 2.1, 2.2, 2.3, 2.4, 2.5, 2.6, 2.7, 2.8. Section 2.1 outlines the testing setup, whereas Section 2.2 details the hardware development of HeartPrinter's components. Section 2.3 describes HeartPrinter's integration in the surgical workflow, and Section 2.4 addresses the taring process for the electromagnetic tracking sensor. Section 2.5 focuses on the introducer mechanism, including its components and deployment of HeartPrinter on the epicardium. Sections 2.6 and 2.7 present two algorithms: one for determining suction base positions and the other for registering an anatomical heart model to the pose of the robot. Finally, Section 2.8 describes the evaluation procedure for the new robot hardware, the introducer mechanism, and the two algorithms.

### 2.1 Testing Model Setup

**FIGURE 3:**
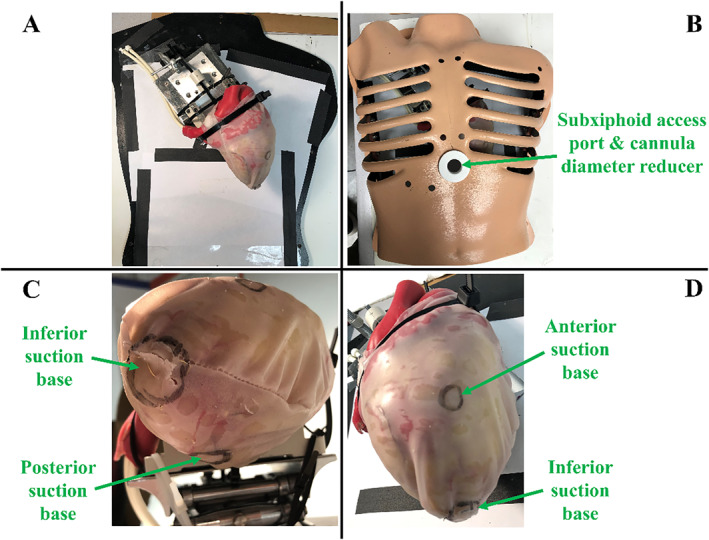
Testing model setup. (A) The rubber heart model mounted to a fixture, and positioned anatomically. The pericardial sac is fitted over the rubber heart. (B) The thorax model is shown, with the diameter reducer added to the subxiphoid access port. The target regions for the following suction bases are labelled on the pericardial sac with a marker: (C) inferior and posterior suction bases and (D) inferior and anterior suction bases.

A rubber beating‐heart model (Model 1008, The Chamberlain Group, Great Barrington, MA, USA) was used for testing the introducer mechanism with the new robot design. The same heart model was used without the beating functionality for evaluating the workspace determination and registration subsystems. When the beating functionality was enabled, the pulse rate was set to 72 beats per minute [23, 32] to simulate a human heart. A standardised heart model was used in this study, so anatomical variability, including differences in ventricular size, wall thickness, and epicardial surface profile, was not assessed. These factors may influence suction base placement and the resulting workspace. However, HeartPrinter's suction base placement methodology and workspace determination algorithm are designed with sufficient flexibility to accommodate typical variations in cardiac anatomy. As shown in Figure 3A,B, the heart was positioned anatomically relative to the thorax (Model 1077, The Chamberlain Group, Great Barrington, MA, USA). The thorax was used to evaluate HeartPrinter's components and the introducer mechanism but not the workspace determination and registration subsystems in this study.

To more realistically simulate operation on a living patient, a diameter‐reducer was added to the subxiphoid access port on the thorax to reduce the diameter of the opening to 20 mm (Figure 3B). A pericardial sac (Model 1112, The Chamberlain Group, Great Barrington, MA, USA) was fitted over the heart to replicate the pericardium, and target regions for the suction bases on the epicardium were marked on the pericardial sac (Figure 3C,D). For some processes, a flashlight was placed within the thorax to increase visibility for imaging. For tests requiring force measurements, a force gauge (Nextech Global Company Limited, Thailand) with a range of 50 N was used.

### 2.2 Hardware Development

#### 2.2.1 Robot Components

**FIGURE 4:**
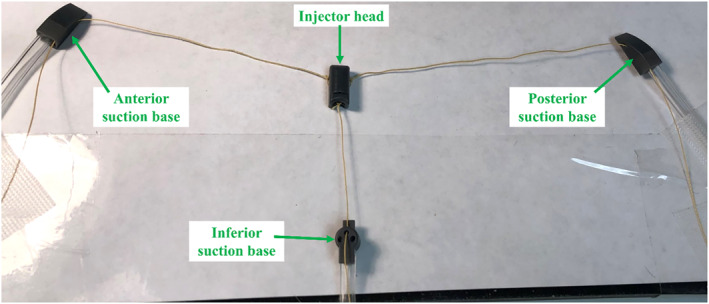
Prototype of HeartPrinter used in this study. The suction base and injector head are shown along with the suction lines and cables.

The prototype of HeartPrinter used in this study is shown in Figure 4, composed of the components, which will be described in the following sections.

##### 2.2.1.1 Superior Suction Bases

**FIGURE 5:**
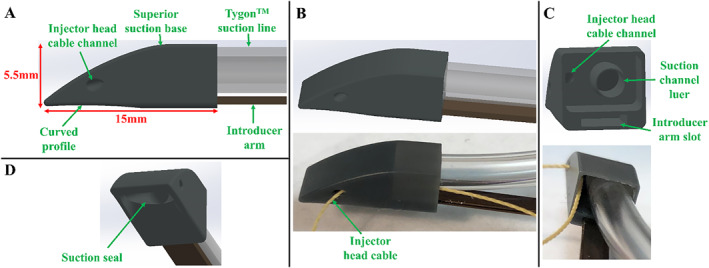
Design and prototype of the superior suction bases. The posterior suction base is shown. (A) Side view of the suction base, which features a curved bottom profile and an injector head cable channel. The Tygon suction line and Nitinol introducer arm are slotted into the suction base. (B) View of the CAD model and prototyped model for the suction base, suction line, and introducer arm. The injector head cable passing through the cable channel is shown on the prototyped model. (C) Second view of the CAD model and prototyped model. The suction line fits over the suction channel luer, the injector head cable passes through the cable channel, and the introducer arm fits into its slot. (D) Suction seal, which interfaces with the heart's surface to form the vacuum seal.

The design of the superior suction bases is shown in Figure 5, with the posterior suction base shown as an example. HeartPrinter has two superior bases: the anterior suction base which is positioned anteriorly on the epicardium, and the posterior suction base which is positioned posteriorly. Deployment of these bases at their target regions defines two of the three points of contact required for HeartPrinter to achieve a stable platform on the heart. Compared to the previous designs [13, 23, 24], the new design of the superior suction bases features several updates. Firstly, as shown in Figure 5A, there is now a smooth top profile in the transition from the distal to proximal end of the suction base, enhancing safety of pericardial contact and enabling greater manoeuvrability in the intrapericardial space with the introducer arm. Moreover, the bottom region of the suction base that is in contact with the heart has a curved profile (Figure 5A), which strengthens the vacuum seal with the epicardium.

As seen in Figure 5A and B, the nitinol introducer arm is slotted into the proximal end of the suction base (Figure 5C), which enables the operator to control the position and orientation of the suction base. Once the suction base is deployed at its target region on the epicardium and suction is activated, the introducer arm can be slid out from the base and removed from the patient. The Tygon suction line (Figure 5A,B) also fits into the proximal end of the suction base, fitting over the suction channel luer fitting (Figure 5C). This luer fitting provides improvements in geometrical matching and maintaining the vacuum seal with the suction line compared to previous circular fitting designs [13, 23, 24]. The suction channel then feeds into the suction seal (Figure 5D), creating an interface with the heart's surface. The suction base also features a channel for the cable to pass from the actuator to the injector head (Figure 5A–C). The posterior suction base is shown in Figure 5, whereas the anterior suction base has a cable channel on the opposite side.

##### 2.2.1.2 Inferior Suction Base

**FIGURE 6:**
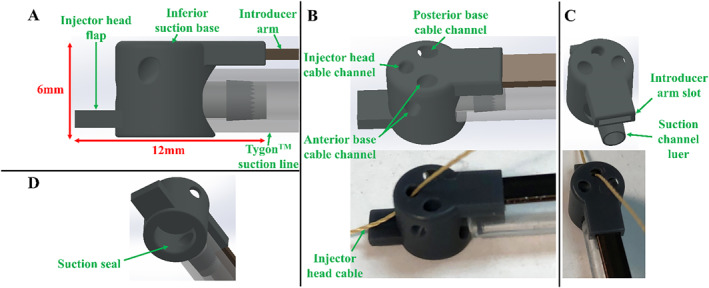
Design and prototype of the inferior suction base. (A) Side view of the suction base, which features a flap for interfacing with the injector head during the insertion process. The Tygon suction line and Nitinol introducer arm are slotted into the suction base. (B) View of the CAD model and prototyped model for the suction base, suction line, and introducer arm. The injector head cable passing through the injector head cable channel is shown in the prototype model. There are also two other channels for the cables from the anterior and posterior suction bases, which is a continued feature from previous designs, but are not used in this study. (C) Second view of the CAD model and prototyped model. The suction line fits over the suction channel luer, the injector head cable passes through the injector head cable channel, and the introducer arm fits into its slot. (D) Suction seal, which interfaces with the heart's surface to form the vacuum seal.

The model of the inferior suction base is shown in Figure 6, which is positioned on the epicardium near the apex of the heart. Once deployed after the superior bases, the three contact points needed for HeartPrinter to achieve a stable platform on the heart are defined. The updated design of the inferior base enhances HeartPrinter's flexibility by not constraining the deployment of the superior bases and improves compatibility with the introducer mechanism. The first feature is a flap (Figure 6A), which fits into the injector head while deploying the suction base near the apex of the heart. This geometrical linking between the injector head and inferior suction base is used to insert them together and determine the position of the inferior base, which will be described later. Similarly to the superior bases, the Tygon suction line and Nitinol introducer arm (Figure 6A,B) are slotted into the proximal end of the suction base at their respective attachment points (Figure 6C).

The second improvement is the independence of the inferior suction base. In previous designs, stiff flexure arms, suction lines, or cables from the superior bases passed through the inferior suction base before exiting the patient [13, 24]. This forced the inferior base to be greater than double its current size and made adherence to the epicardium challenging, particularly when the anterior or posterior cables were actuated, thereby pulling the inferior base off the heart. In this design, the anterior and posterior cable channels (Figure 6B) are unused, and no components from the superior bases pass through the inferior base. This allows the inferior base to be deployed and operated independently from the superior bases. Like the superior bases, the inferior base includes a channel for the injector head cable to pass through (Figure 6C), and a suction seal to interface with the heart (Figure 6D).

##### 2.2.1.3 Injector Head

**FIGURE 7:**
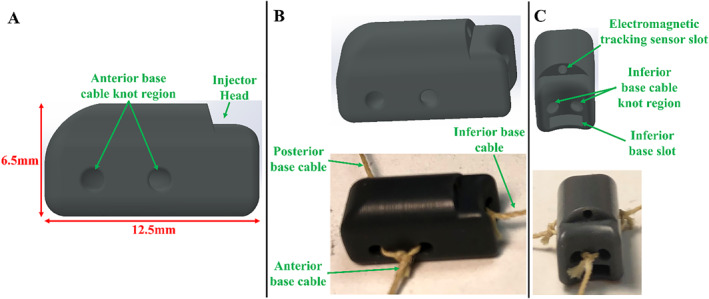
Design and prototype of the injector head. The injection system is not yet incorporated. (A) Side view of the injector head with cleats for the cables from each of the bases. (B) View of the CAD model and the prototyped model for the injector head. The cables from each of bases are attached to the injector head. (C) Second view of the CAD model and prototyped model. Slots for a 6‐DOF electromagnetic tracking sensor and the inferior base flap are shown, as well as the inferior base cable cleat region.

The injector head, shown in Figure 7, features three cleats for the cables from the suction bases (Figure 7A,B). During HeartPrinter's deployment, the flap on the inferior suction base slots into the injector head's slot (Figure 7C), allowing joint insertion. The injector head also includes a slot for a 6‐degree‐of‐freedom (DOF) electromagnetic tracking sensor (3D Guidance, Northern Digital Inc., Waterloo, ON, Canada) (Figure 7C). The sides of the injector head are geometrically matched to the suction bases, enabling the sensor to determine their positions, as described later.

The actuator cables are made from high‐strength twisted Kevlar, which has a strength‐to‐weight ratio approximately five times greater than steel. While the cable deformation is not zero, it is minimal at the force scale seen by HeartPrinter, and therefore, hysteresis is expected to contribute only negligibly to positional errors of the injector head.

#### 2.2.2 Tabletop Setup

The tabletop instrumentation, positioned alongside the patient during the procedure, is shown in Figure 2. It includes a motor control hub, three Dynamixel AX‐12A motors (ROBOTIS, Seoul, South Korea) with spools for cable actuation, three DYZL‐107 load cells (DAYSENSOR, Bengbu, China) with transmitters, a data acquisition device for measuring cable tension, and a power supply. Suction is provided to the suction bases and the dilating suction tool through flexible suction lines connected to a PowerVac Aspirator suction pump (Precision Medical, Northampton, PA). Cable guides at the edge of the top platform direct the cables from the load cells to the subxiphoid access port (Figure 3B). The platform height exceeds that of the thorax model (Figure 3B) to facilitate suction base positioning with the introducer mechanism. The tabletop setup is designed to be placed by the patient's right side, with the cable guides positioned closest to the patient.

### 2.3 Surgical Workflow

**FIGURE 8:**
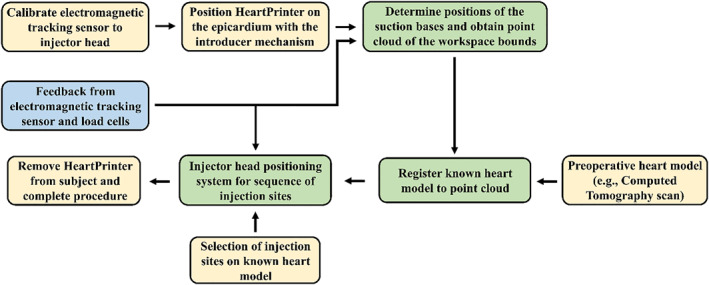
Surgical workflow for HeartPrinter. Processes in green are primarily system controlled, and processes in yellow are primarily operator controlled. The system begins by calibrating the electromagnetic tracking sensor placed inside the injector head, followed by positioning the robot on the epicardium with the introducer mechanism. This is followed by determination of HeartPrinter's workspace, registration and positioning of the injector head at various injection sites. Throughout the procedure, global position feedback is received from the electromagnetic tracking sensor, and feedback for tension in the cables is received from the load cells.

The surgical workflow for HeartPrinter, shown in Figure 8, is divided into preoperative, intraoperative, and postoperative stages. In the preoperative stage, a heart model, for example obtained from a computed tomography scan, is prepared as a reference for the procedure. The intraoperative stage begins with calibrating the 6‐DOF electromagnetic tracking sensor inside the injector head. The introducer mechanism then deploys the suction bases and injector head onto the epicardium. Once deployed, the tracking sensor identifies the positions of the suction bases relative to the electromagnetic tracking transmitter, and the preoperative heart model is registered to the points captured by the tracker. With the heart model registered, the operator selects injection sites, and the positioning system moves the injector head sequentially to each site to deliver injections. Throughout this stage, the system continuously receives global position feedback from the tracker and cable tension feedback from the load cells. In the postoperative stage, the operator safely removes HeartPrinter by deactivating all suction and retrieving the cables of the robot.

### 2.4 Electromagnetic Tracking Sensor Taring

Prior to positioning the suction bases and injector head on the epicardium with the introducer mechanism, the 6‐DOF electromagnetic tracking sensor must be tared to calibrate its orientation measurements. This step ensures two key objectives: enabling the operator to place the sensor in the injector head at any orientation without manually zeroing the roll, pitch, and yaw angles, and aligning these angles with the tracking sensor's orientation in the injector head, which is critical for estimating the positions of the suction bases later in the workflow.

The tracking sensor is first inserted into its slot on the injector head, which is positioned in front of the electromagnetic tracking transmitter's front hemisphere. The distal end of the injector head is aligned with the positive *y*‐axis of the transmitter. For this study, the injector head was taped flat to a shared table with the transmitter. Future developments could include a dedicated taring jig to precisely align the injector head with the transmitter on a flat surface. Once the injector head is positioned with respect to the transmitter, the roll, pitch and yaw angles of the tracking sensor are zeroed, thereby aligning the sensor's orientation with the transmitter. After taring, the tracking sensor must remain securely fixed within the injector head throughout the procedure.

### 2.5 Introducer Mechanism

#### 2.5.1 Design Requirements

##### 2.5.1.1 Size and Shape

A subxiphoid approach is used to insert HeartPrinter with the introducer mechanism through an accessory device such as a cannula. Previously used cannulas had an inner diameter of 20 mm [13]. Once inserted, HeartPrinter is guided towards the pericardium near the apex of the heart. For this, the introducer mechanism and HeartPrinter's components are designed to fit through such accessory devices. As mentioned previously, testing in this study performed on the thorax model is done with a diameter reducer of 20 mm (Figure 3B).

##### 2.5.1.2 Approach Mechanism

The angle of approach of the first components for the introducer mechanism used to pass through the skin incision should be angled superficially at approximately 20° to the horizontal plane and superiorly to the immediate left of the left shoulder, ensuring that the musculophrenic and superior epigastric arteries are avoided [33, 34]. A superficial angle during initial advancement is critical for averting deeper abdominal components; however, it should not be too superficial to avoid puncturing the internal mammary artery [34]. Once over the diaphragm, the angle can be increased to approximately 45° for a posterior approach towards the fibrous pericardium [33].

##### 2.5.1.3 Ease of Use

Once epicardial access is achieved with the introducer mechanism, access should be maintained through the same point of entry on the pericardium and into the pericardial space for deploying the suction bases at their target regions. This approach eliminates the need for repeated navigation from the skin to the pericardial space for each suction base, reducing procedure time and minimizing the risk of patient injury. This process must ensure consistent access while providing the necessary manipulability and flexibility to reach the suction base target regions anteriorly, posteriorly, and at the apex of the heart.

#### 2.5.2 Primary Components

The following devices are the key components for the introducer mechanism presented.

##### 2.5.2.1 Tuohy Needle

**FIGURE 9:**
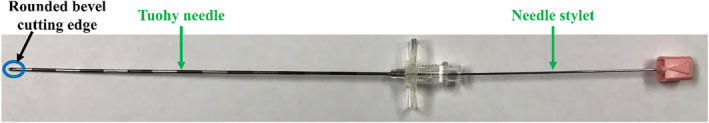
18G by 6 in. Tuohy needle. The needle features a rounded bevel cutting edge at the distal end. This allows for the needle to be advanced with the bevel pointed upward and away from the cardiac border to decrease the risk of injury. The needle also comes with a stylet, which prevents clogging of the needle's lumen, while navigating the needle through surrounding anatomy when approaching the pericardium.

The 18G 6‐in. Tuohy needle (Epimed, Dallas, TX, USA), shown in Figure 9, is used to establish the entry point through the pericardium and into the pericardial space, while also aiding with navigation from the skin to the pericardium. The needle's distal end features a rounded bevel cutting edge, which enables advancement with the bevel pointed upward and away from the cardiac border, reducing the risk of injury. Moreover, a stylet that fits within the needle is used to prevent clogging of the needle's lumen during navigation through surrounding anatomy. Use of a Tuohy needle for epicardial access is a common procedure [33, 34], making it a suitable choice for this application.

##### 2.5.2.2 Dilating Suction Tool

**FIGURE 10:**
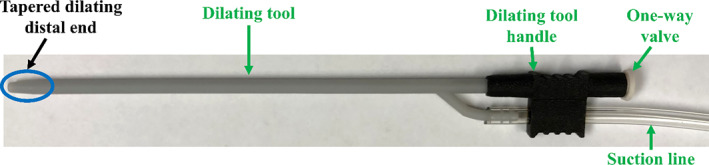
Prototype of the dilating suction tool. The device provides suction ability through its lumen, which allows the pericardium to be pulled away from the epicardium prior to penetrating through the pericardium with the Tuohy needle. The distal end is tapered to dilate the point of entry through the pericardium. A one‐way valve at the proximal end ensures that suction can be maintained at the distal end, and allows passage of complimentary devices, specifically the Tuohy needle.

**FIGURE 11:**
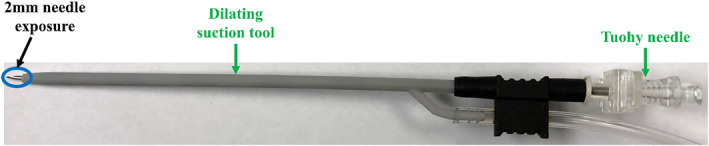
Passage of the Tuohy needle through the dilating suction tool. The Tuohy needle is inserted at the proximal end of the dilating suction tool, through the one‐way valve. The Tuohy needle can be inserted until 2 mm exposure of the needle tip is achieved at the distal end, which occurs when the Tuohy needle handle makes contact with the proximal end of the dilating suction tool.

The prototype dilating suction tool, which has an outer diameter of 3.4 mm, is shown in Figure 10. This device serves two primary functions. First, it creates separation between the pericardium and epicardium prior to penetration with the Tuohy needle by using suction to pull the pericardium away. Suction is delivered through the lumen of the device via a suction line integrated into the handle, utilizing the same suction line as for the suction bases. A one‐way valve at the proximal end maintains suction at the distal end, while permitting passage of the Tuohy needle. The second function of the tool is to dilate the opening in the pericardium using its tapered distal end. While the width of pericardial space cannot typically be consistently determined due to uneven pericardial fluid distribution [35], the dilating suction tool ensures 2 mm of Tuohy needle exposure at the distal end (Figure 11), facilitating penetration of the pericardium, which is typically just under 2 mm thick [36].

##### 2.5.2.3 Steerable Guide Tool

**FIGURE 12:**
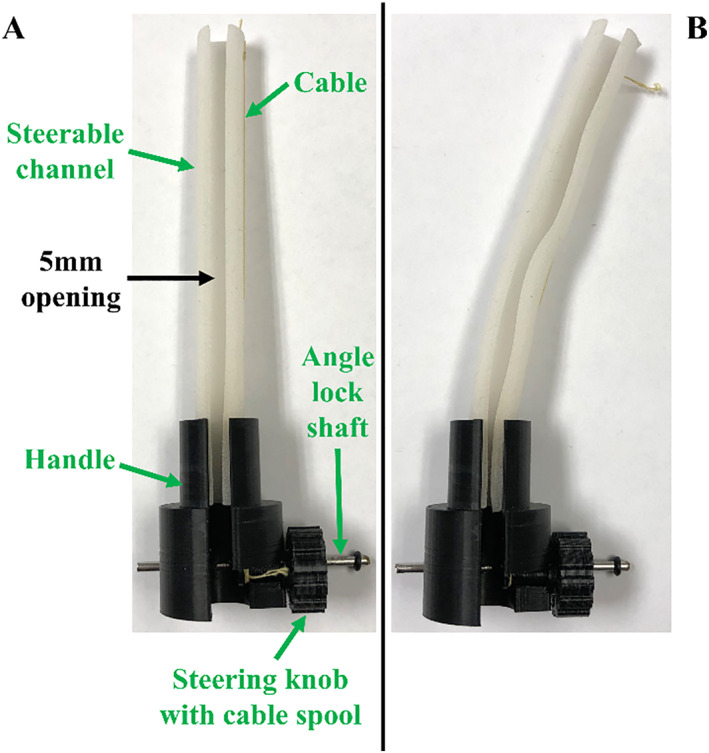
The prototype of the steerable guide tool is shown in the (A) straight configuration and (B) curved configuration. The device features a flexible channel body fitted into a handle, with a cable passing along the length of the channel body. The cable is knotted at the distal end of the channel body, and is wrapped around a cable spool placed within the handle at the proximal end. Rotation of the steering knob causes spooling of the cable, thereby causing deflection of the steerable channel. The angle lock shaft is used to fix the steerable guide tool to a set a curvature. The 5 mm opening prevents premature exit of the suction bases and injector head, which are 6 mm wide, and allows the steerable guide tool to be passed over the dilating suction tool.

The steerable guide tool prototype is shown in Figure 12. This tool is directed over the dilating suction tool through the point of entry on the pericardium, and is used to guide the superior suction bases to their target regions on the epicardium. The guide consists of a body with a flexible channel that is fitted into a handle at the proximal end. Within the handle, a steering knob is connected to a cable spool, with the cable running along the length of the channel and terminating in a knot at the distal end. Rotation of the steering knob causes the cable to spool, resulting in the deflection of the steerable channel from a straight configuration (Figure 12A) to a curved one (Figure 12B). The angle lock shaft is then engaged to fix a set curvature of the channel.

When the steerable guide is within the pericardial space, it can be curved and rotated to align the distal end with the target region of the suction base, either anteriorly or posteriorly. The suction base can then be inserted through the steerable guide tool until it exits the distal end and is directed to its target region. The 5‐mm opening in the guide ensures that the 6‐mm wide superior suction bases are inserted sequentially without premature exit out of the channel before reaching the distal end, while also allowing the injector head cable, suction line and introducer arm to be released from the steerable guide tool once the suction base reaches its target. The opening also permits the steerable guide tool to pass over the dilating suction tool.

#### 2.5.3 Mechanism Description

##### 2.5.3.1 Epicardial Access

**FIGURE 13:**
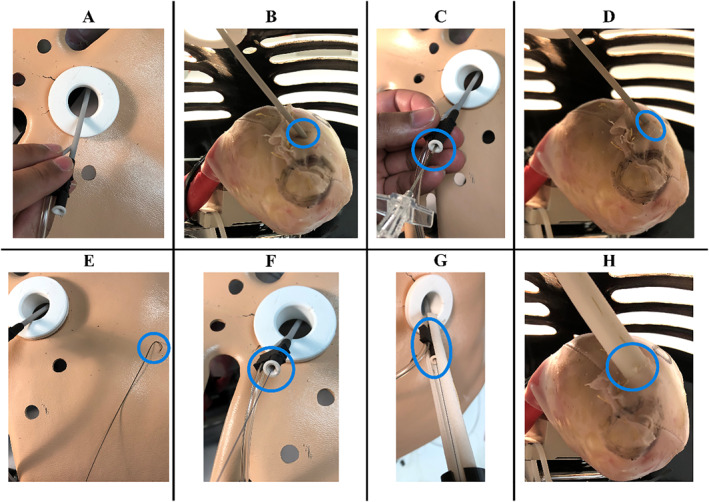
Workflow for gaining and maintaining epicardial access with the introducer mechanism. (A) The dilating suction tool is inserted through the 20 mm opening, and targeted towards the pericardium near the apex of the heart. The Tuohy needle with its stylet can be used with the dilating suction tool to facilitate navigation through surrounding anatomy. (B) The dilating suction tool contacts the pericardium near the apex of the heart. Suction is then activated to pull the pericardium away from the epicardium. (C) The Tuohy needle is inserted through the dilating suction tool in order to penetrate the pericardium, which is achieved with gentle pressure coupled with respiratory movement of the heart. (D) The dilating suction tool is pushed distally over the tip of the Tuohy needle in order to enter and dilate the pericardial opening, and the Tuohy needle is removed. The dilating suction tool is now in the pericardial space. (E) A J‐tip guidewire is used to maintain epicardial access. (F) The J‐tip guidewire is inserted through the dilating suction tool and into the pericardial space. (G) The steerable guide tool is inserted over the dilating suction tool towards the pericardial opening. (H) The steerable guide tool is in the pericardial space.

The workflow for gaining and maintaining epicardial access is shown in Figure 13. First, the dilating suction tool is inserted through the 20‐mm opening (Figure 13A) with suction disabled. The entry point on the skin is typically 2–3 cm below and slightly lateral to the xiphoid process [33]. To navigate through subcutaneous tissue, the Tuohy needle with its stylet can be inserted through the dilating suction tool. As per the design requirements, the tool is angled at approximately 20° to the coronal plane and superiorly to the immediate left of the left shoulder [33, 34]. After crossing the diaphragm, the angle of the suction tool can be increased to approximately 45° for a posterior approach towards the fibrous pericardium [33]. Once near the fibrous pericardium, the Tuohy needle and stylet, if used, are removed from the suction tool.

Once the suction tool makes contact with the fibrous pericardium (Figure 13B), suction is activated to separate the fibrous and parietal pericardial layers from the visceral pericardial layer, also referred to as the epicardium. The Tuohy needle was then reinserted through the suction tool without its stylet (Figure 13C). As the needle tip approaches 2 mm of exposure at the distal end, gentle pressure paired with respiratory movement of the heart typically suffices to penetrate the parietal pericardial layer [33]. As the needle passes through the pericardial layers, tenting of the parietal layer may occasionally occur [33], and a tactile ‘give’ is felt by the operator as the needle enters the pericardial space [33, 34].

After the Tuohy needle has entered the pericardial space, suction on the dilating suction tool is disabled, and the tool is advanced distally over the Tuohy needle tip. This inserts the suction tool into the pericardial space and dilates the opening formed by the Tuohy needle (Figure 13D). To maintain epicardial access in the pericardial space, a 0.014‐in. J‐tip guidewire (Figure 13E) was inserted through the dilating suction tool and into the pericardial space (Figure 13F), with the J‐curve securing access through the opening. Finally, the steerable guide tool was inserted over the dilating suction tool and guidewire towards the pericardial opening (Figure 13G), further dilating the opening until the guide tool reached the pericardial space (Figure 13H).

##### 2.5.3.2 Posterior Suction Base Positioning

**FIGURE 14:**
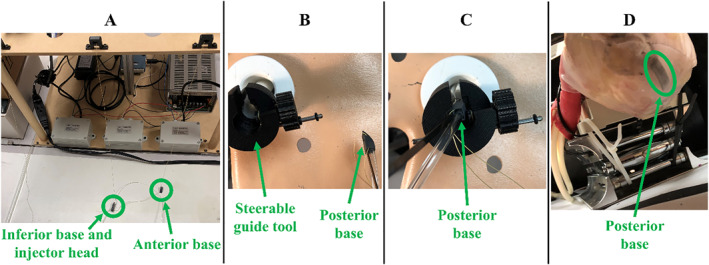
Workflow for positioning the posterior suction base at its target region on the epicardium. (A) The cable for the posterior base is released by its actuator, allowing the posterior base to be pulled towards the patient. The inferior base, injector head, and anterior base are held near the tabletop setup. (B) After the steerable guide tool is rotated and steered in the appropriate direction for posterior access, the posterior base is oriented for insertion through the steerable guide tool. (C) The posterior base is inserted through the steerable guide tool. (D) The posterior base sliding posteriorly along the epicardium is shown after exiting the distal end of the steerable guide tool.

With the steerable guide tool in the pericardial space, the posterior suction base is deployed first at its target region (Figure 14). From the tabletop setup, the cable for the posterior base is released by its actuator, allowing the posterior base to be pulled towards the subxiphoid access port via the introducer arm. Meanwhile, inferior suction base, injector head and anterior suction base remained near the tabletop setup (Figure 14A). Inside the pericardial space, the guide tool was then rotated to direct the posterior suction base appropriately for posterior access, with the 5‐mm opening facing towards the patient's left side (Figure 14B). The posterior base is then inserted through the steerable guide tool (Figure 14C) until it exits the distal end of the guide tool and slides posteriorly along the epicardium (Figure 14D). Once the posterior suction bases reaches its target region, suction is turned on and the introducer arm is removed from the suction base and patient.

##### 2.5.3.3 Anterior Suction Base Positioning

**FIGURE 15:**
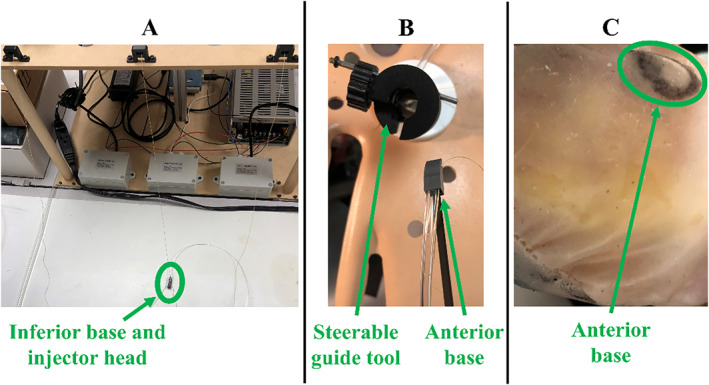
Workflow for positioning the anterior suction base at its target region on the epicardium. (A) The cable for the posterior base is released by its actuator, allowing the anterior base to be pulled towards the patient. The inferior base and injector head are held near the tabletop setup. (B) After the steerable guide tool is rotated and steered in the appropriate direction for anterior access, the anterior base can be inserted through the steerable guide tool. (C) The anterior base sliding anteriorly along the epicardium is shown after exiting the distal end of the steerable guide tool.

The mechanism for positioning the anterior suction base is shown in Figure 15. First, the actuator releases the cable for the anterior base, while the injector head and inferior suction base remain near the tabletop setup (Figure 15A). Once the anterior suction base reaches the subxiphoid access port, the guide tool is rotated and steered in the appropriate direction for anterior access, with the 5‐mm opening oriented towards the patient's right side (Figure 15B). The anterior suction base is then inserted through the guide tool, sliding anteriorly along the epicardium to its target region (Figure 15C). Upon reaching its target, suction is activated and the introducer arm is retracted from the base and patient.

##### 2.5.3.4 Inferior Suction Base and Injector Head Positioning

**FIGURE 16:**
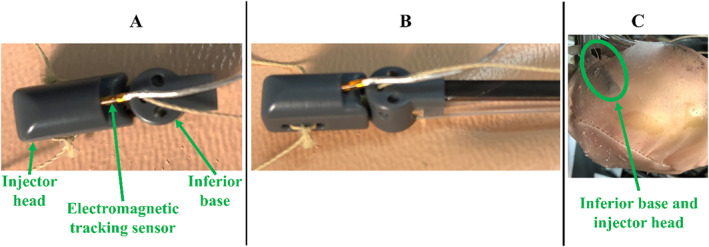
Workflow for positioning the inferior suction base and injector head near the apex of the heart. (A) To position the inferior suction base and injector head together, the injector head is slotted over the inferior base's flap. (B) The cables from the superior bases are pulled with their actuators to direct the inferior suction base and injector head towards the subxiphoid access port. Once in close proximity to the access port, the introducer arm and suction line are attached to the inferior base. (C) Using the cables of the superior bases as guides and with their gradual spooling, the injector head and inferior base are directed towards the pericardial opening for positioning at the apex.

The inferior suction base and injector head are deployed together near the apex of the heart, as shown in Figure 16. Firstly, the steerable guide is removed from the pericardial opening, with epicardial access maintained by the cables from the superior bases which connect to the injector. As shown in Figure 16A, the injector head is slotted over the inferior suction base's flap. The cables from the superior bases are then pulled by their actuators, directing the inferior suction base and injector head towards the access port. When in close proximity to the access port, the nitinol introducer arm and suction line were attached to the inferior base (Figure 16B). Next, with gradual spooling of the superior‐base cables and using them as guides, along with manipulation of the introducer arm by the operator, the injector head and inferior suction base are directed through the pericardial opening and towards the apex (Figure 16C). As with the superior bases, the introducer arm is retracted from the inferior base and patient once the inferior base reaches its target.

### 2.6 Suction Base Position Determination

In this study, position determination of the suction bases was performed on a static heart without beating and the thorax. This is the first study analyzing HeartPrinter in three dimensions on a heart model, with a pericardial sac and with suction adherence of the suction bases. Future work will adapt this method for operation on a beating heart through the subxiphoid access port [37].

#### 2.6.1 Inferior Base

**FIGURE 17:**
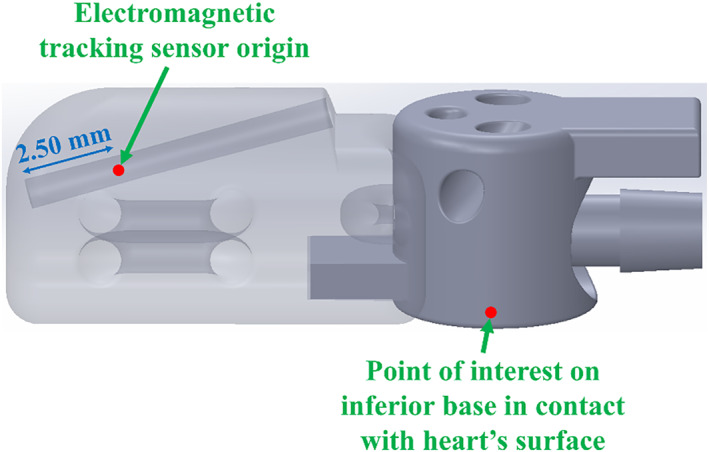
Visualization of the offset distance from the electromagnetic tracking sensor origin within the injector head to the point of interest on the inferior suction base that is in contact with the heart's surface.

The position of the inferior suction base with respect to the electromagnetic tracking transmitter is determined only when the injector head is slotted over the inferior base's flap and is determined as follows. First, the offset distance from the electromagnetic tracking sensor origin within the injector head to the point of interest on the inferior suction base is pre‐determined from the computer‐aided design (CAD) model. This offset aligns with the coordinate frame of the electromagnetic tracking transmitter, positioned in front of the front hemisphere. The point of interest on the inferior suction base is defined as the centre point of the suction seal that contacts the surface of the heart. A visualization of this offset distance between the sensor origin and this point of interest is shown in Figure 17.

After deploying the inferior base and injector head on the epicardium using the introducer mechanism, 100 data points of coordinates and Euler angles are collected by the electromagnetic tracking sensor at that single position. The average of these data points is used to compute a rotation matrix based on the average Euler angles, as shown in Equation (1). To ensure compatibility with the electromagnetic tracking system's application programming interface, a clockwise *xyz* Euler angle convention is adopted to define the rotation matrix, as shown in Equation (1). The angles about the *z*, *y*, and *x* axes of the sensor are referred to as azimuth (*a*), elevation (*e*), and roll (*r*), respectively.

(1) $$R=R_{x}\left(r\right)R_{y}\left(e\right)R_{z}\left(a\right)=\left(\begin{array}{ccc} 1 & 0 & 0\\ 0 & \cos\;r & \sin\;r\\ 0 & -\sin\;r & \cos\;r \end{array}\right)\left(\begin{array}{ccc} \cos\;e & 0 & -\sin\;e\\ 0 & 1 & 0\\ \sin\;e & 0 & \cos\;e \end{array}\right)\left(\begin{array}{ccc} \cos\;a & \sin\;a & 0\\ -\sin\;a & \cos\;a & 0\\ 0 & 0 & 1 \end{array}\right)$$

Using the average coordinates of the sensor, the offset distance and the rotation matrix, the position of the point of interest on the inferior suction base can be calculated with respect to the electromagnetic tracking transmitter. This is expressed in Equations ((2), (3), (4)) given a 3 × 3 rotation matrix, *R*, where *R*(*n,m*) is the element of the *n*th row and *m*th column.

(2) $$x_{\text{inferior}\_\text{base}}=x_{\text{sensor}}+x_{\text{offset}}\cdot R\left(1,1\right)+y_{\text{offset}}\cdot R\left(2,1\right)+z_{\text{offset}}\cdot R\left(3,1\right)$$

(3) $$y_{\text{inferior}\_\text{base}}=y_{\text{sensor}}+x_{\text{offset}}\cdot R\left(1,2\right)+y_{\text{offset}}\cdot R\left(2,2\right)+z_{\text{offset}}\cdot R\left(3,2\right)$$

(4) $$z_{\text{inferior}\_\text{base}}=z_{\text{sensor}}+x_{\text{offset}}\cdot R\left(1,3\right)+y_{\text{offset}}\cdot R\left(2,3\right)+z_{\text{offset}}\cdot R\left(3,3\right)$$

#### 2.6.2 Superior Bases

The position of the anterior suction base is determined as follows. The offset distance from the electromagnetic tracking sensor origin within the injector head to the point of interest on the anterior suction base is calculated preoperatively from the CAD model. This offset distance is calculated assuming that the injector head is adjacent to the anterior suction base, with the right face of the anterior base aligned to the left face of the injector head. After determining the position of the inferior suction base, the actuator for the anterior suction base cable is engaged to pull its cable, while the other two actuators passively rotate in the opposite direction. This manoeuvre releases the injector head from the inferior suction base flap and directs it towards the injector head cable channel on the anterior suction base.

As the injector head translates towards the anterior suction base, it traverses along the outer boundary of the triangular robot workspace. During this motion, *xyz* points and Euler angles are collected using the electromagnetic tracking sensor for the registration phase of the workflow. Moreover, as the injector head approaches the anterior suction base, tension in the anterior base cable increases. A predefined tension threshold is used to determine when the injector head is immediately adjacent to the anterior suction base, stopping the spooling of the anterior base cable when this threshold is met. With the injector head and anterior suction base now aligned and adjacent to each other, the position of the point of interest on the anterior suction base is determined similarly to the inferior suction base, using the average coordinates of the sensor, the offset distance, and the rotation matrix.

This process is then repeated for the posterior suction base by translating the injector head towards the posterior base, and adding the *xyz* points and Euler angles of the trajectory to the set of points for registration. Finally, the injector head is translated back towards the inferior suction base, and these trajectory points and angles are similarly included in the set of points for registration. Using the collected registration points, the transformation from the electromagnetic tracking sensor origin within the injector head to the injector head's point of interest on the heart surface is applied, following the same model equations used for the inferior suction base (Equations ((1), (2), (3), (4))). The point of interest on the injector head is defined as its contact point with the heart, aligned with the *z*‐axis of the sensor origin. Thus, the positions of all suction bases on the heart surface are now determined, and a collection of points which define the outer boundary of the triangular workspace for HeartPrinter can be used for registration.

### 2.7 Registration

In this study, registration of the anatomical heart model constructed preoperatively to the pose of the robot is assumed to be on a static heart without beating and the thorax. Notably, this is the first investigation of HeartPrinter in three dimensions on a heart model with a pericardial sac and vacuum adherence of the suction bases. Future advancements will extend this method to a beating heart [38], operating via a subxiphoid access port.

#### 2.7.1 Registration Workflow

As seen in Figure 8, a preoperative heart model is first input into the registration system. This study uses a three‐dimensional object file generated from a scan of the rubber heart as input. Along with the three‐dimensional heart model, the point cloud defining the outer bounds of the triangular workspace, collected during the suction base position determination phase, is input to the registration system. The registration framework then proceeds as follows. First, on a display, the operator performs a coarse alignment by visually matching the approximate operating region on the heart model (the left ventricular epicardium) to the workspace point cloud. This manual alignment reduces large rotational discrepancies between the point sets, which can otherwise cause global registration methods that rely on nearest‐neighbour correspondences to converge to suboptimal local minima [39, 40]. In this study, both the initial visual alignment and the subsequent global registration were performed using CloudCompare (v2.13.beta, 2023). After global registration, fine alignment was carried out using the Iterative Closest Point (ICP) algorithm, which is widely used for point cloud registration due to its simplicity and robustness [39]. Following ICP convergence, the positions of the injector head and suction bases could be visualized on the registered heart model.

### 2.8 Experimental Test Procedures

In this study, the number of trials for each test was determined by balancing the challenges of repetition with the need to maintain the reliability of the test results.

#### 2.8.1 Suction Bases—Suction Performance

**FIGURE 18:**
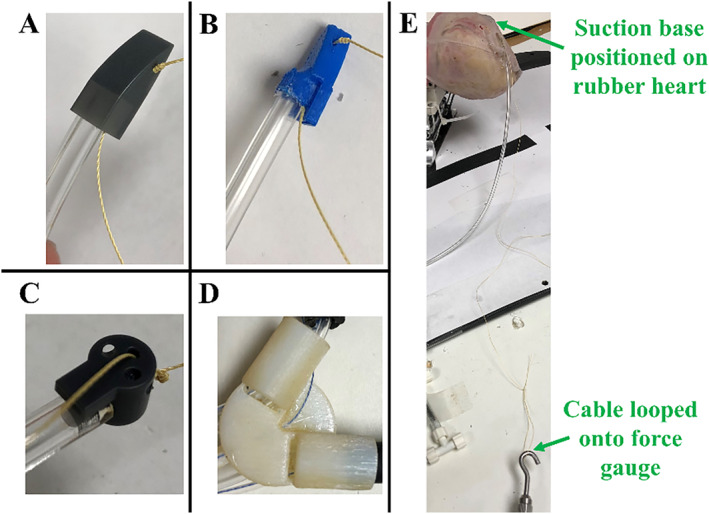
Set‐up for suction performance testing for the suction bases. A cable is knotted through the injector head cable channel, and a suction line is attached to (A) new anterior base, (B) old anterior base and (C) new inferior base. (D) Old inferior base prototype, which could not achieve suction on the rubber heart model and was not tested. (E) Anterior suction base positioned at its target region on the rubber heart model without the beating functionality. The cable was looped onto the force gauge and pulled to record the force before dislodging of the suction base. Testing of the new inferior suction base was performed with it being positioned at the apex of the heart.

The new suction base design was assessed for suction ability against the old prototypes [23]. Five trials (*n =* 5) were performed for each of the two anterior bases (Figure 18A,B) and for the new inferior base (Figure 18C). The old inferior base (Figure 18D) was not tested due to its inability to achieve suction on the rubber heart model. As seen in Figure 18E for the anterior suction base, a cable was knotted at the suction base through the injector head cable channel and was looped onto the force gauge. A suction line was then attached to the suction base, and the base was positioned on the rubber heart model at its target region without the beating functionality. Once full vacuum adherence of the suction base to the rubber heart was achieved, the cable was gradually pulled with the force gauge, simulating the cable pulling by the tabletop actuators. The peak force before the suction base dislodged from the heart was recorded as the minimum dislodgement force. A two‐sample *t*‐test was used to compare the suction ability of the superior suction bases, with results considered significant if *p* < 0.01.

#### 2.8.2 Suction Bases—Introducer Arm Retraction Forces

The forces required to retract the introducer arm from the suction bases were measured. As described earlier, the introducer arm is removed from the suction base once the base adheres to its target region on the epicardium. Thus, the required retraction forces must be less than the minimum dislodgement force of the suction base from the heart, which is critical for assessing the design of the introducer arm slot. As the introducer arm slot is identical in the superior and inferior bases and the suction dislodge forces for both bases were already collected, the test was only conducted on a superior suction base. A cable was first knotted around the proximal end of the Nitinol introducer arm and looped onto the force gauge. The suction base was fixed on a table with tape, and the introducer arm was inserted into its slot on the suction base. Five trials (*n = 5*) were performed by gradually pulling the cable proximally with the force gauge and recording the force when the introducer arm was retracted from the suction base. Two‐sample *t*‐tests were used to compare the introducer arm retraction forces to the suction dislodge forces of the new superior and inferior suction base designs, with results considered significant if *p* < 0.01.

#### 2.8.3 Epicardial Access Performance Testing

##### 2.8.3.1 Tuohy Needle Insertion Forces

Force measurements for the insertion of the Tuohy needle through the pericardium using a subxiphoid approach were collected both with and without the use of the dilating suction tool. A 3D printed coupling was used to attach the Tuohy needle to the force gauge for measurements. Ten measurements (*n =* 10) without use of the dilating suction tool were collected to simulate a typical epicardial access procedure that only uses a Tuohy needle [33, 34], while 10 measurements (*n =* 10) with the suction tool were used for comparison. A two‐sample *t*‐test was used to compare results with and without the suction tool, and results were considered significant if *p* < 0.01.

##### 2.8.3.2 Dilating Suction Tool—Suction and Dilation Performance

The dilating suction tool was first assessed for suction ability on the pericardium. A 3D‐printed coupling was used to attach the dilating suction tool to the force gauge for measurements. Through the access port, the suction tool was inserted towards the pericardial sac at an angle mimicking that of the introducer mechanism procedure described previously with the Tuohy needle held inside it. Once full vacuum adherence of the dilating suction tool to the pericardial sac was achieved with the Tuohy needle inside, the dilating suction tool was pulled with the force gauge. Ten trials (*n =* 10) were performed to capture the force required to separate the tool from the pericardial sac.

The dilating suction tool was then assessed for dilation forces through the pericardium. The same 3D‐printed coupling was used to attach the dilating suction tool to the force gauge. In the heart model, an 18G needle was used to create perforations on the pericardial sac. The dilating suction tool was then placed on a perforation and inserted through the pericardial sac to dilate the opening. The peak force when the dilating suction tool penetrated the pericardial sac was recorded. Ten perforations on the pericardial sac were created for 10 trials (*n =* 10) with the dilating suction tool.

#### 2.8.4 Superior Suction Base Navigation Ability

As navigation of the superior bases within the pericardial space and along the epicardium using the introducer arm is critical for successful positioning of HeartPrinter at the epicardial target regions, navigation ability of the new superior base design was assessed against the previous superior suction base design. A test setup was developed using a foam mat and nylon sock, with the sock fitted snugly over the foam mat to simulate a worst‐case scenario of the pericardial sac pressing down on the epicardium. The edges of the mat were clamped down to a table. Both new (Figure 18A) and old (Figure 18B) superior suction bases were manipulated by their respective introducer arms and inserted at the entry point. The suction base was then slid along the mat to the exit point. Three trials (*n =* 3) were performed for each suction base, and navigation ability was visually recorded.

#### 2.8.5 Suction Base Positioning Accuracy

Accuracy in positioning of the suction bases at their target regions was assessed using a 6‐DOF electromagnetic tracking sensor. With the electromagnetic tracking transmitter fixed adjacent to the thorax, desired position coordinates were pre‐defined for each of the targets on the epicardium of the heart model without beating. Then, the introducer mechanism was used to position the suction bases posteriorly, anteriorly and at the apex of the heart during beating. Once positioned on the epicardium, the beating of the heart was stopped and the electromagnetic tracking sensor was used to determine their actual positions. Positional error of the actual position relative to the desired position was calculated as the average root mean square error (RMSE) for each target region across five trials (*n =* 5). The coordinate frame for this test was defined with the positive *y*‐axis oriented towards the patient's left shoulder, the positive *z*‐axis oriented downwards towards the patient's posterior, and the positive *x*‐axis derived using a right‐handed coordinate frame.

#### 2.8.6 Suction Base Position Determination Accuracy

The accuracy of placement of the suction bases was assessed using two 6‐DOF electromagnetic tracking sensors after HeartPrinter was deployed with the introducer mechanism. The first sensor was used to find the true positions of the suction bases on the rubber heart model, while the second sensor was placed inside the injector head and was used to determine the calculated positions via the suction base position determination algorithm. Positional errors of the calculated positions relative to the true positions were measured as average RMSE for each of the suction bases across five trials (*n =* 5), along with the standard deviation to assess consistency. The coordinate frame for this test was identical to that described in Section 2.8.5.

#### 2.8.7 Assessment of Registration Alignment Error

Using the five point clouds obtained from the suction base position determination accuracy test, the three‐dimensional heart model was registered to each point cloud. The alignment error between the transformed points of the heart model and the corresponding points from the outer bounds point clouds was quantified as average RMSE in the *x*, *y* and *z* axes across five trials (*n =* 5). Additionally, the average fitness for the five trials was recorded, where fitness represents the division of the number of inlier correspondences by the total number of points in the respective triangular outer bounds point cloud. The corresponding point in an outer‐bound point cloud, associated with a point in the transformed heart model, is defined as the closest point within a specified distance threshold. The coordinate frame for this test was identical to that described in Section 2.8.5.

## 3 Results

The Results section is organized into Sections 3.1, 3.2, 3.3, 3.4, 3.5, 3.6, 3.7, 3.8, which correspond to Section 2.8 in the Materials and Methods. These subsections present results on the new robot hardware, the introducer mechanism, and the two algorithms.

### 3.1 Suction Bases—Suction Performance

The mean force required to dislodge the superior suction base was 2.6 ± 0.2 N for the new design and 2.2 ± 0.1 N for the old design. The associated *p* value was 0.003, indicating a statistically significant improvement in suction performance of the new superior base design. The mean dislodgement force for the new inferior base prototype at the apex of the heart was 1.1 ± 0.1 N. As noted previously, the test could not be conducted on the old inferior base.

### 3.2 Suction Bases—Introducer Arm Retraction Forces

Mean introducer arm retraction forces from a superior suction base are summarized in Table 1, alongside previously collected suction base dislodge forces. The average introducer arm retraction force was 0.8 ± 0.1 N. Both superior and inferior suction base dislodge forces were significantly greater than the introducer arm retraction forces (*p* < 0.0001 and *p* < 0.0014, respectively).

**TABLE 1:** Suction base dislodge forces from the rubber heart as a measure of suction strength for the new designs compared to the introducer arm retraction forces.

Suction base type	Suction base dislodge force [mean ± SD]	Introducer arm retraction force [mean ± SD]	*p* value
Superior	2.6 ± 0.2	0.8 ± 0.1	< 0.0001
Inferior	1.1 ± 0.1		0.0014

### 3.3 Tuohy Needle Insertion Forces

The average force required to insert the Tuohy needle through the pericardial sac on the rubber heart model was 1.2 ± 0.2 N without the dilating suction tool and 1.5 ± 0.2 N with the dilating suction tool. Insertion of the Tuohy needle with the dilating suction tool was performed only after achieving adequate suction on the pericardium. The difference in mean forces was statistically significant (*p* = 0.007), yet it was deemed acceptable, as explained further in the Discussion section.

### 3.4 Dilating Suction Tool—Suction and Dilation Performance

The average force required to pull the dilating suction tool away from the pericardial sac was 1.8 ± 0.1 N, measured after the dilating suction tool achieved full suction adherence on the pericardial sac while held at an angle, with the Tuohy needle inside it. This force, which exceeded the Tuohy needle insertion forces with the dilating suction tool collected previously, confirms that the pericardial sac can be securely held by the suction tool during needle insertion. The mean force for dilating perforations by the suction tool in the pericardial sac in the rubber heart model was 5.5 ± 1.4 N.

### 3.5 Superior Suction Base Navigation Ability

**FIGURE 19:**
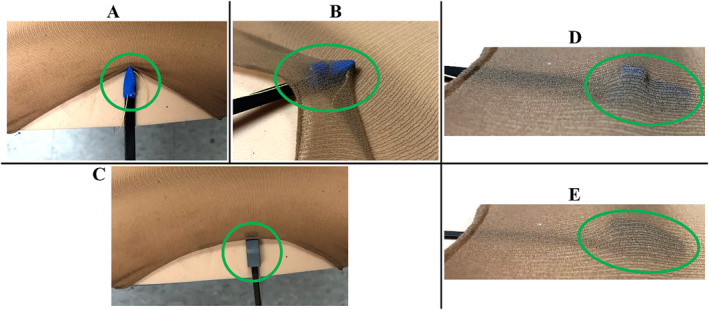
Navigation ability assessment of the old and new superior suction base designs in the nylon sock setup. (A) Insertion of the old superior suction base at the point of entry. (B) Front edge of the old superior suction base caught on the nylon sock. (C) Insertion of the new superior suction base at the point of entry. Guidance of the old superior suction base along the foam mat and towards the exit point for the (D) old superior suction base, and (E) new superior suction base.

The navigation ability of the new superior suction base exceeded that of the old design. Upon insertion at the entry point, the front edge of the old superior suction base caught onto the nylon sock, pulling the sock forward, as seen in Figure 19A,B. In contrast, the new superior suction entered smoothly without catching on the sock (Figure 19C). While sliding along the foam mat towards the exit point, the circular suction line fitting of the old superior suction base repeatedly caught onto the nylon sock (Figure 19D), whereas the new superior suction base navigated smoothly within the environment (Figure 19E).

### 3.6 Suction Base Positioning Accuracy

Positioning accuracy of the suction bases at their target regions on the epicardium of the rubber heart model is shown in Table 2. The average RMSE in the *x*, *y* and *z* axes was 9.36 mm for the anterior base, 13.51 mm for the posterior base, and 9.96 mm for the inferior base. The largest positional errors were for the posterior base in the *x* and *z* directions, measuring 9.44 and 9.32 mm, respectively. Across all bases, the greatest positional errors occurred along the *x*‐axis.

**TABLE 2:** Average accuracy for positioning the suction bases at their target regions on the epicardium of the rubber heart model without beating.

Suction base	*n*	Average RMSE			
		*x* [mm]	*y* [mm]	*z* [mm]	Total [mm]
Anterior	5	6.53	4.56	4.92	9.36
Posterior		9.44	2.56	9.32	13.51
Inferior		7.11	4.91	4.95	9.96

### 3.7 Suction Base Position Determination Accuracy

The accuracy of determining the positions of the suction bases on the epicardium of the rubber heart model using the position determination algorithm is shown in Table 3. The average RMSE in the *x*, *y* and *z* axes was 3.07 mm for the anterior base, 3.73 mm for the posterior base, and 1.45 mm for the inferior base. Position determination accuracy error for both superior suction bases was more than double the error for the inferior suction base. Additionally, position determination accuracy error was the greatest in the *x*‐axis for all bases.

**TABLE 3:** Average accuracy for determining the positions of the suction bases on the epicardium of the rubber heart model without beating using the position determination algorithm.

Suction base	*n*	Average RMSE				Standard deviation		
		*x* [mm]	*y* [mm]	*z* [mm]	Total [mm]	*σ*_*x*_ [mm]	*σ*_*y*_ [mm]	*σ*_*z*_ [mm]
Anterior	5	2.03	1.20	1.96	3.07	1.36	0.718	1.50
Posterior		2.32	2.22	1.90	3.73	1.75	1.06	1.67
Inferior		1.08	0.732	0.633	1.45	0.877	0.724	0.557

To assess the consistency of these errors, the standard deviation of the positional error in each axis is also shown in Table 3. The anterior and posterior bases had the highest variation in the *x* (*σ*_*x*_anterior_ = 1.36 mm, *σ*_*x*_posterior_ = 1.75 mm) and *z* axes (*σ*_*z*_anterior_ = 1.50 mm, *σ*_*z*_posterior_ = 1.67 mm), while the inferior base demonstrated the most consistent results overall, with standard deviations below 0.9 mm in all axes.

### 3.8 Assessment of Registration Alignment Error

**FIGURE 20:**
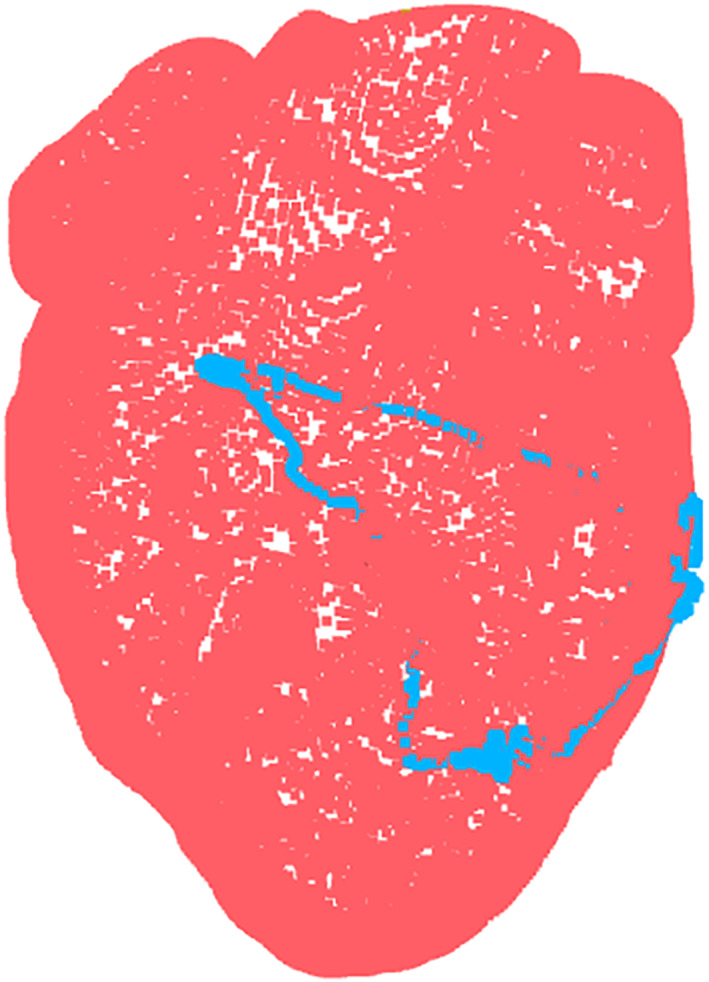
Registration visualization of the heart model to one of the point cloud trials collected from the outer workspace at the left side of the heart.

The registration error between the transformed heart model and the point clouds of the outer workspace bounds is presented in Table 4. The average RMSE for the five trials was 1.14 mm in the *x*‐axis, 1.02 mm in the *y*‐axis and 0.829 mm in the *z*‐axis, with an average fitness of 0.103. A visual representation of the heart model registered to one of the point cloud trials from the outer workspace is shown in Figure 20.

**TABLE 4:** Mean registration error between the transformed heart model and the point clouds of the outer workspace bounds.

*n*	Average fitness	Average RMSE			
		*x* [mm]	*y* [mm]	*z* [mm]	Total [mm]
5	0.103	1.14	1.02	0.829	1.74

## 4 Discussion

The developments to HeartPrinter's components for adhering to and operating on the epicardium provide improved stability on the heart's surface and better compatibility with the introducer mechanism. Given a bottom curved profile for the suction bases and the suction line being fitted over a luer fitting instead of a circular fitting, the vacuum seal with the epicardium was strengthened, as demonstrated by the increase in mean suction force compared to the previous design. This enhancement ensures a stable platform for HeartPrinter to compensate the heartbeat and respiratory motions of the heart, reducing the risk of complications and increased procedural delays. To further improve suction base stability on the surface of the heart, future designs should eliminate tangling of the suction line and cable for each suction base. Currently, tangling can dislodge the suction line from the base or pull the base away from its target region. A potential solution is a bi‐lumen flexible tube integrated into the suction base, with one lumen for suction and the other for the cable.

Although suction‐base adherence in this study was evaluated on an artificial beating heart, prior work with HeartPrinter and HeartLander has demonstrated robust performance in vivo on porcine models and ex vivo on ovine hearts. These studies reported minimal fluid aspiration and reliable suction performance [13, 32]. Patronik et al. further examined epicardial fat effects, showing that although some fat may enter the suction chamber, it does not clog the line or reduce traction [41]. Traction decreases only on fully exposed adipose tissue, a condition unlikely to be frequently encountered by HeartPrinter.

For compatibility with the introducer mechanism, the force required to retract the introducer arm from the suction bases was less than the suction adherence force, ensuring that the introducer arm can be removed from the bases once they adhere to their target regions on the epicardium. Navigation within the pericardial sac was also enhanced for the superior suction bases when operated with the introducer arm. The improved suction base design, featuring a smooth top profile transitioning from the distal to proximal end of the suction base, allowed for significantly smoother sliding within the constrained environment compared to the previous design. Not only does this enhance the user experience but also minimizes patient safety risks such as excessive insertion forces and damage to the suction bases. Furthermore, HeartPrinter's tabletop setup was fully redesigned to ensure functionality with the introducer mechanism and the components it operates.

The redesigned introducer mechanism met the design requirements for size, shape, approach mechanism, and ease of use. HeartPrinter's footprint was reduced to fit through a 20‐mm cannula by reducing the size of the inferior suction base by over 50%, geometrically matching the injector head's flap for simultaneous insertion of the inferior suction base and the injector head and eliminating components passing through the inferior base. To meet the approach mechanism requirement, the dilating suction tool was manipulated to avoid puncturing critical anatomy before reaching the fibrous pericardium and navigated through subcutaneous tissue with the Tuohy needle and its stylet. For ease of use, the J‐tip guidewire sufficiently maintained access through the pericardial opening, and with the steerable guide tool inserted over it to dilate the opening and maintain its position in the pericardial sac, this process was repeatable for the suction bases. Future reductions in the size of the steerable guide tool could further enhance HeartPrinter's minimal invasiveness and streamline the introducer mechanism workflow.

The Tuohy needle insertion force with the dilating suction tool was greater than without the tool; however, this was deemed acceptable, because suction from the dilating tool pulls the pericardium up against the Tuohy needle. This creates a free‐standing, moveable thin base for puncture, requiring greater manipulation of the Tuohy needle and, consequently, more force compared to when the pericardial sac is held stationary against the epicardium without the suction tool. Although the suction tool successfully pulled the pericardium away from the epicardium, minimizing the risk of puncture to the epicardium, the full suction ability of the dilating suction tool was not maintained during puncture. This was likely due to leakage at the proximal end out of the valve during Tuohy needle movement and contaminants on the pericardial sac that clogged the dilating suction tool. Stability of the puncture site and suction retention can be enhanced in future designs by using a suction pump with higher vacuum pressure and increasing the internal diameter of the dilating suction tool. This would provide a larger gap around the Tuohy needle, increasing the cross‐sectional area for contact. The taper at the distal end should be retained to ensure smooth insertion during dilation and minimize the risk of excessive force, as demonstrated by the measured dilation forces through pericardial sac perforations in this study.

Successful positioning of the suction bases at the target regions with the introducer mechanism was demonstrated. Since the target regions serve as general references rather than for high‐precision absolute positioning, a positioning error of 9–13 mm for each base was sufficient to establish a workspace covering the full left ventricular wall. The posterior base exhibited the greatest positional error due to a larger error in the *z*‐axis. This error was caused by movement of the heart model during positioning, as the guide tool and introducer arm applied a downward force on the heart, with the bottom half of the heart freely suspended and unsupported by the fixture. Improved fixturing may reduce this artefact in future studies; however, this deflection is an inherent compliance property of the rubber heart that cannot be fully resolved. This deflection is not expected in vivo, where the heart is supported by pericardial and surrounding tissues. Positional errors were the greatest in the *x*‐axis as the introducer arm slightly pushed the heart superiorly within the fixture, and limited visualization of the suction bases during positioning as only the subxiphoid access port could be seen. In a clinical setting, fluoroscopy and radiopaque suction bases would enable correction of these errors during placement.

HeartPrinter is intended to enable gene therapy delivery, which requires numerous injections over a broad region of the left ventricular epicardium to achieve adequate vector distribution [10]. The robot's cable‐driven design allows rapid and repeated injector head positioning within the triangular workspace defined by the suction bases, supporting dense injection patterns over a large epicardial area. As this study focused on establishing this workspace through benchtop verification, a detailed analysis of injection distribution relative to clinical coverage requirements was beyond the scope. This will be undertaken once the complete injection system is integrated and evaluated in vivo.

The suction base position determination algorithm and registration framework represent the first such workflows for HeartPrinter on a heart model with a pericardial sac and vacuum adherence of the suction bases, utilizing an electromagnetic tracking sensor. For the suction base position determination algorithm, the injector head successfully reached each suction base beneath the pericardial sac, enabling estimation of suction base locations and defining the outer bounds of the workspace. This also provided the collection of points needed for registration. The larger and less consistent errors observed for the superior suction bases likely result from two factors: the inferior base is measured immediately after deployment without movement of the injector head, minimizing heart displacement, whereas movement towards the superior bases introduces higher cable tensions and contact forces that can shift the suction base or slightly deflect the heart within the fixture. Future work will refine cable tension thresholds and injector head approach speeds to reduce this variability.

The registration framework provided an effective procedure for aligning the heart model to the point clouds that defined the outer bounds of HeartPrinter's triangular workspace with an average error of less than 2 mm. The use of CloudCompare for global registration mimicked how the operator would generally align the heart model on a display, and ICP enabled further registration refinement. As shown in Figure 20, a good portion of the left side of the heart is defined by HeartPrinter's triangular workspace, allowing the operators to select specific injection sites within it. In the future, the confidence levels of the triangular workspace point clouds can be increased by having the injector head go to each suction base multiple times, thereby densifying the point cloud, reducing noise, and increasing the registration fitness.

Electromagnetic tracking was used in this study only to obtain HeartPrinter's injector head positions in a controlled benchtop setup. No metallic cardiac implants were present, and therefore, electromagnetic field distortion along with dynamic tracking errors were not encountered. Comparative evaluation of alternative sensing modalities, for example fibre Bragg gratings, and assessment of electromagnetic performance in the presence of cardiac metal or cardiac motion represent important future steps but were outside the scope of this study.

Ensuing design improvements and integration for HeartPrinter will focus on implementing the injection system and heartbeat compensation in a three‐dimensional environment, which will require space‐time localisation and registration. For registration, transitioning from a static to a beating heart introduces the challenge of incorporating temporal registration alongside spatial registration [38]. This involves modelling the periodic motion of the surface of the heart, where the preoperative image represents a snapshot of the heart at a specific physiological phase [38]. These advancements will enable more accurate assessments by better representing the heart and cardiothoracic environment while also providing further validation of the designs and workflows presented in this study.

## 5 Conclusion

This study demonstrates significant developments in HeartPrinter's hardware and software infrastructure. These include components for epicardial adhesion and operation and their compatibility with the introducer mechanism, refinement of the introducer mechanism for precise robot deployment, and preliminary workspace determination and registration workflows on a heart model with a pericardial sac and vacuum adherence of the robot's suction bases. By meeting functional design requirements of the robot and the introducer mechanism, HeartPrinter can be positioned on the epicardium safely and repeatedly and stably adhere to the beating heart. Moreover, utilisation of the electromagnetic tracking sensor in the injector head facilitates intraoperative determination of HeartPrinter's workspace and registration of a preoperative three‐dimensional heart model.

## Author Contributions

Conceptualization and design: Aman Ladak. Data collection: Aman Ladak. Data analysis: Aman Ladak. Writing – original draft preparation: Aman Ladak. Writing – reviewing: Cameron N. Riviere, Johannes O. Bonatti, Roger J. Hajjar, and Alaaeldin A. Shalaby. Writing – editing: Aman Ladak. Clinical insight: Johannes O. Bonatti, Roger J. Hajjar, and Alaaeldin A. Shalaby. Supervision: Cameron N. Riviere. Funding acquisition: Cameron N. Riviere and Aman Ladak. Final approval: All authors have read and approved the final version of the manuscript.

## Funding

This research was partially supported by the U.S. National Heart, Lung, and Blood Institute (Grant R01HL174945), and Carnegie Mellon University's GSA/Provost GuSH Grant.

## Ethics Statement

The authors have nothing to report.

## Conflicts of Interest

C. N. Riviere reports holding equity in HeartLander Surgical, Inc. and is a co‐inventor of U.S. patent US10736703B2, held by Carnegie Mellon University. The authors report no other conflicts of interest in this work.

## Permission to Reproduce Material

This manuscript does not contain any reproduced material from other sources that requires permission.

## Data Availability

The data that support the findings of this study are available from the corresponding author upon reasonable request.
